# Myor/ABF-1 Mrna Expression Marks Follicular Helper T Cells but Is Dispensable for Tfh Cell Differentiation and Function *In Vivo*

**DOI:** 10.1371/journal.pone.0084415

**Published:** 2013-12-26

**Authors:** Delphine Debuisson, Nathalie Mari, Sébastien Denanglaire, Oberdan Leo, Fabienne Andris

**Affiliations:** Laboratoire d'Immunobiologie, Université Libre de Bruxelles, Gosselies, Belgium; INSERM-Université Paris-Sud, France

## Abstract

Follicular T helper cells (Tfh) are crucial for effective antibody responses and long term T cell-dependent humoral immunity. Although many studies are devoted to this novel T helper cell population, the molecular mechanisms governing Tfh cell differentiation have yet to be characterized. MyoR/ABF-1 is a basic helix-loop-helix transcription factor that plays a role in the differentiation of the skeletal muscle and Hodgkin lymphoma. Here we show that MyoR mRNA is progressively induced during the course of Tfh-like cell differentiation *in vitro* and is expressed in Tfh responding to Alum-precipitated antigens *in vivo*. This expression pattern suggests that MyoR could play a role in the differentiation and/or function of Tfh cells. We tested this hypothesis using MyoR-deficient mice and found this deficiency had no impact on Tfh differentiation. Hence, MyoR-deficient mice developed optimal T-dependent humoral responses to Alum-precipitated antigens. In conclusion, MyoR is a transcription factor selectively up-regulated in CD4 T cells during Tfh cell differentiation *in vitro* and upon response to alum-protein vaccines *in vivo*, but the functional significance of this up-regulation remains uncertain.

## Introduction

Upon activation, naive CD4^+^ T helper cell (Th) precursors can differentiate into functionally distinct T cell lineages including Th1, Th2, Th17, and follicular T helper (Tfh) cells. Among the critical signals that direct the induced patterns of gene expression in maturing helper T cell subsets are cytokine-induced specific transcription factors. IL-12 drives Th1 cell differentiation through the activation of the transcription factors STAT4 and T-bet [1], [2]. IL-4 induces Th2 cell differentiation through the actions of STAT6 and GATA-3 [3], [4], whereas the development of Th17 cell is prompted by a combination of IL-6 plus TGFβ and requires expression of STAT3 and RORγt [5].

Follicular T helper cells are key regulators of germinal center (GC) formation and T cell-dependent long-term humoral immunity [6], [7]. First defined as CD4^+^ T cells located in human tonsillar GCs [8], Tfh cells express high levels of chemokine receptor 5 (CXCR5), inducible T cell co-stimulator (ICOS) and programmed cell death 1 (PD1), that are associated with a downregulation of the T cell zone-homing receptor CC-chemokine receptor 7 (CCR7) and IL-2 receptor-α (IL-2Rα) [9]. Tfh cells also express B- and T-lymphocyte attenuator (BTLA), CD40L and the cytoplasmic adaptor protein signal lymphocyte activation molecule (SLAM)-associated protein (SAP) [6]. These molecules are important for the migration of Tfh lymphocytes to B cell follicles and for the provision of signals leading to initiation and maintenance of B cells GC responses [10], [11]. Several cytokines, including IL-6 and IL-21, have been shown to drive Tfh cell differentiation by promoting the activation of STAT3 and expression of the transcriptional repressor BCL6, considered as the critical regulator of Tfh cell development *in vivo* [12]–[17], although additional transcription factors (including basic leucine zipper transcriptional factor ATF-like (BATF), interferon-regulatory factor 4 (IRF4) and C-Maf), also have important regulatory functions for Tfh cell differentiation [6].

The main cytokine-signature of Tfh cells is IL-21, which provides crucial signals to B cells leading to antibody production, memory and plasma cell differentiation [18]–[20], although CD4 T cells that are not Tfh cells, including Th17 cells, can express substantial IL-21 [21].

Despite intensive studies devoted to this novel T helper cell population, there are still gaps in our understanding of how Tfh cells are induced *in vitro* and *in vivo* [22]. To get insight into the specific transcription factors that operate during Tfh differentiation, we undertook a detailed transcriptomic analysis of cells stimulated *in vitro* in the presence of exogenous IL-6, a procedure previously shown to induce the development of Tfh-like cells [14]. This analysis led us to identify MyoR/ABF-1, a member of the basic helix-loop-helix (b-HLH) transcription factor family as a gene whose expression is induced in naive T cells upon differentiation into Tfh-like cells.

Proteins of the b-HLH family are required for a number of different developmental pathways, including neurogenesis, lymphopoiesis, myogenesis and sex determination [23], [24]. MyoR/ABF-1 is coded by the *musculin* (msc) gene and has been independently identified in mouse skeletal muscle precursors (MyoR for Myogenic Repressor [25]–[27], and in Hodgkin lymphomas and Epstein-Barr virus-transformed B-cell lines (ABF-1, Activated B cell Factor-1 [28]–[30]. In B cell lymphomas, ABF-1 heterodimerizes with the E2A proteins and is implicated in inhibition of the E2A-dependent B cell transcription program [28]. Hence, overexpression of ABF-1 in B-cell lines reduced B-cell-specific gene expression, leading to reprogramming of neoplastic B cells in Hodgkin lymphomas [29]. Similarly, MyoR has been shown to form heterodimers with E proteins that bind the same DNA sequence as myogenic bHLH/E protein heterodimers, and acts as a potent transcriptional repressor that blocks myogenesis and activation of E-box-dependent muscle genes [25].

MyoR-KO mice were generated by the team of E. Orson [27]. These mice were born at the expected Mendelian ratios and had no evident abnormalities, except that specific facial muscles were absent in mice lacking both MyoR and capsulin [27]. However, the functional role of MyoR in T lymphocytes has not been clarified.

The objective of the current work is to assess whether the expression of MyoR is associated with Tfh cells differentiated both *in vitro* and *in vivo* and to evaluate its putative role in Tfh cell development.

## Results

### The mRNA coding for MyoR is highly expressed in Tfh-like cells and its expression is regulated by STAT3

**Figure 1:**
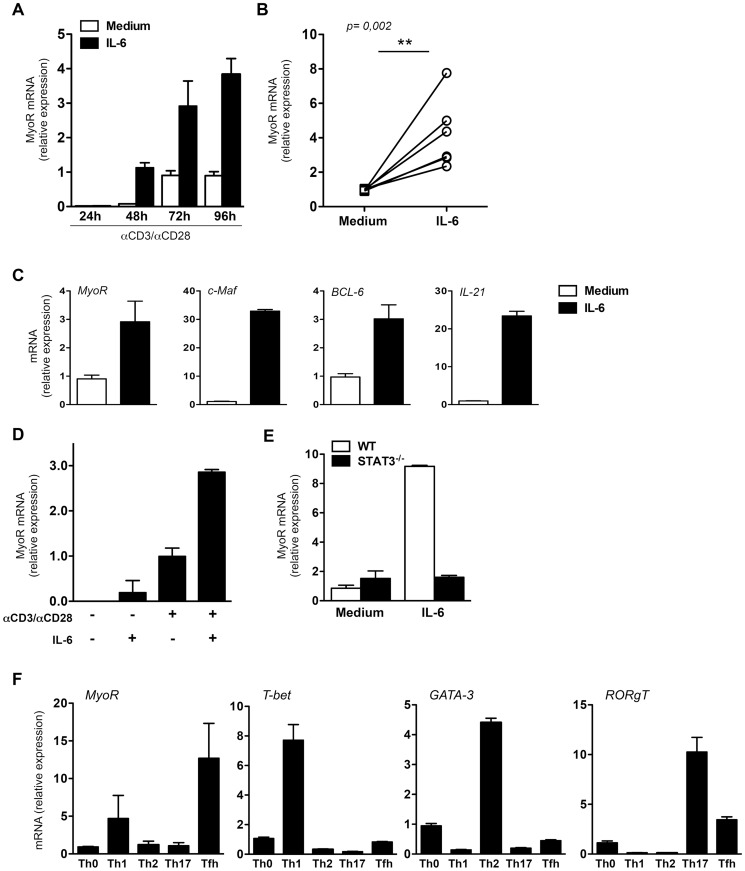
Tfh-like cells express MyoR mRNA. Naive CD62L^+^CD4^+^ T cells purified from the spleen of C57BL/6 mice were stimulated with plastic-coated anti-CD3 and anti-CD28 mAbs under neutral conditions (medium) or in the presence of IL-6 (Tfh-like condition). Expression level of the indicated genes was assessed by quantitative RT-PCR and expressed as relative expression to RPL32 mRNA. (A) Kinetic expression of MyoR under Th0 and Tfh culture conditions; (B) Compilation of individual experiments showing increased MyoR expression in 72 h cultured-Tfh-like cells; (C) Expression of a set of Tfh-associated genes in 72 h-cultured cells in the presence of IL-6; (D) MyoR expression in resting versus TcR activated, IL-6-treated T lymphocytes; (E) Expression of MyoR in 48 h-Tfh-like activated wild type and STAT3-deficient T cells; (F) MyoR, T-bet, GATA-3 and RORγT mRNA expression in 72 h-polarized Th0, Th1, Th2, Th17 and Tfh-like cells. The 72 h activated-Th0 condition (48 h in panel E) was set to 1. Histograms represent the mean ± SD of duplicates and are representative of three independent experiments (A, C-E and F-panels T-bet, GATA-3, RORγT) or the mean ± SD of three independent experiments (F-panel MyoR). Dots in panel B represent individual paired Th0 and Tfh cultures data sets. Differences between groups in B were analyzed with the Mann-Whitney test for 2-tailed data. * p<0.05; n.d. =  not detectable

A comparative microarray analysis performed on *in vitro* stimulated murine CD4^+^ T cells led to the identification of a subset of mRNAs, including MyoR-encoding mRNA, whose expression was elevated in cells stimulated in the presence of IL-6 (see Table S1 for complete description of the microarray data). To confirm this observation, naive CD4^+^ T cells isolated from C57BL/6 mice were activated with anti-CD3 and anti-CD28 antibodies in the presence and absence of IL-6. MyoR mRNA expression was assessed after 24, 48, 72 and 96 h using real-time PCR. As shown in Figure 1A, MyoR mRNA gradually accumulated in activated cells, a response that was accelerated and reinforced in the presence of IL-6 (Figure 1A, B). The Tfh-like features of IL-6-treated cells was confirmed by higher expression of mRNA coding for BCL-6 [31], IL-21 [17], [32] and c-Maf [33], [34], compared to cells activated in the absence of IL-6 (medium condition, Figure 1C).Addition of IL-6 in the absence of receptor stimulation failed to induce significant levels of MyoR mRNA (Figure 1D) suggesting a role for TcR-initiated signals in the induction of MyoR gene transcription.

We next examined the levels of MyoR mRNA in other *in vitro* derived-T helper cell subsets. After a 3 day-activation under standard Th1, Th2, Th17 or Tfh polarizing conditions (see methods section for details), cells were analyzed for master transcription factors (T-bet, GATA-3 and RORγt to confirm polarization) and MyoR mRNA expression. As shown in Figure 1F, MyoR mRNA expression appeared to be higher in the Tfh-like cells, compared to the other subsets, under these *in vitro* stimulation settings.

However, despite a reproducible induction of MyoR mRNA under Tfh culture conditions, we were unable to detect the MyoR protein in Tfh-like cells, using a commercially available antibody reagent (see Figure S1 for western blotting and control experiment). Further experiments will be required to conclude if the absence of protein detection results from a transient MyoR expression or from an expression level of MyoR below the detection limit of the assay.

STAT3 is an important transcription factor for Tfh differentiation activated in response to IL-6 [12], [14]. Induction of MyoR mRNA was completely abrogated in STAT3-deficient T cells activated in the presence of IL-6 (Figure 1E). Moreover, IL-21, another STAT3-activating cytokine also promoted MyoR expression in anti-CD3/CD28 activated naive Th cells (Figure S2).

Collectively, these data suggest that MyoR mRNA is expressed in T helper cells in response to TcR stimulation and its expression is further increased following activation of the IL-6/STAT3 signaling pathway.

### In vivo-induced Tfh cells express MyoR mRNA

**Figure 2:**
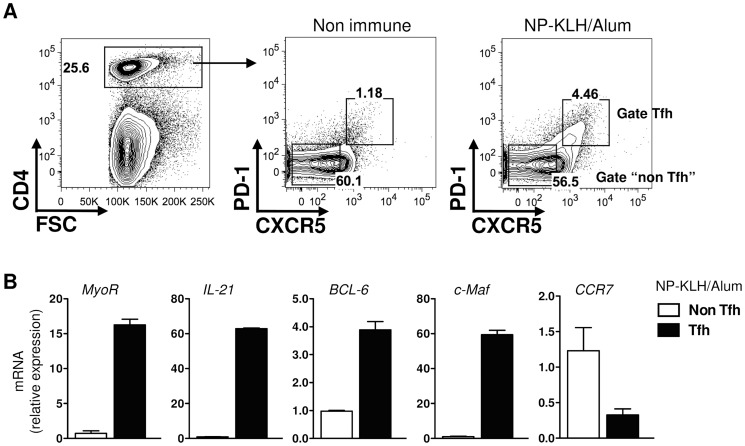
MyoR mRNA is expressed in Tfh cells induced *in vivo*. Tfh and non Tfh cells from NP-KLH/Alum immunized mice were tested for relative MyoR mRNA expression. (A) Representative flow cytometric plot showing the percentage of Tfh cells in non-immune and NP-KLH/Alum treated mice and the gating strategy for sorted Tfh and non-Tfh cells from immune mice on day 7. (B) Relative expression of MyoR and selected genes in sorted Tfh and non Tfh cells. Histograms represent the mean ± SD of duplicates and are representative of three independent experiments.

Within 72 h of immunization a subset of the responding CD4 T cells acquires the expression of CXCR5 and migrates into the B follicles. These migrant follicular T helper cells have been described both in humans and mice and are characterized by their high levels of expression of CXCR5, PD1 and ICOS receptors [8], [35]–[37]. To test if MyoR is expressed in Tfh cells *in vivo*, a group of mice was immunized against KLH/Alum. Seven days later, CD4^+^CXCR5^+^PD1^+^ (Tfh) cells and CD4^+^CXCR5^−^PD1^−^ (non-Tfh) cells were sorted from the spleen of immunized mice (Figure 2A). As expected, sorted Tfh cells expressed high levels of CXCR5 and PD1, but also ICOS, compared to non-Tfh cells, confirming the efficacy of the gating strategy (data not shown). Real-time RT-PCR confirmed an increased expression of MyoR mRNA in Tfh cells compared to non-Tfh cells (Figure 2B, left panel), in agreement with the previous *in vitro* analysis. Note that the identity of Tfh cells was also confirmed by the elevated expression of BCL6, C-Maf and IL-21 mRNA, accompanied by the decreased expression of CCR7 mRNA (Figure 2B).

### Naive T helper cells from MyoR^−/−^ mice develop into Tfh-like cells in vitro

**Figure 3:**
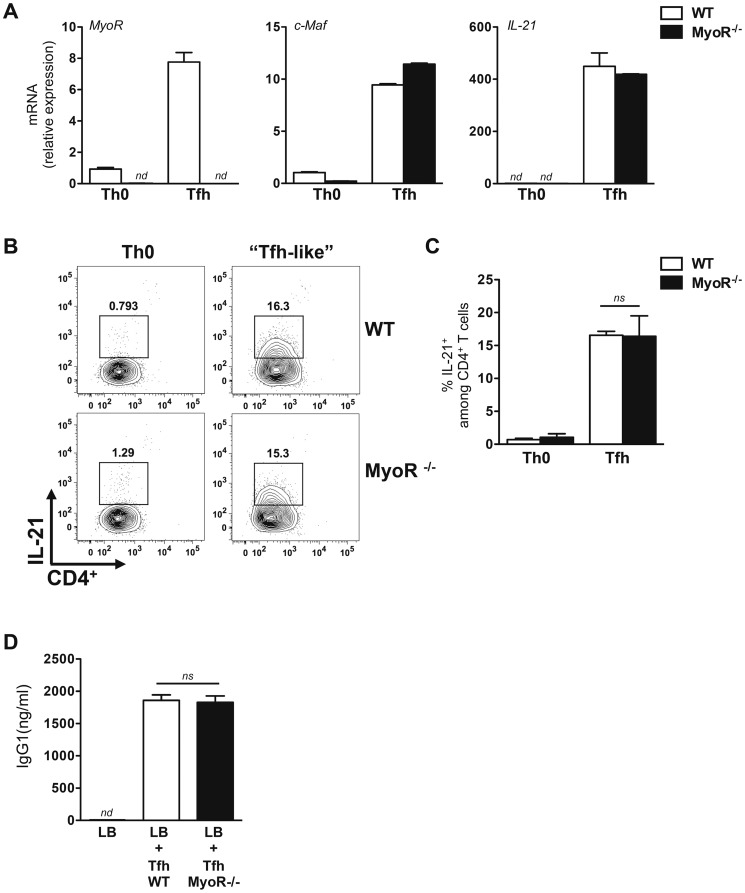
MyoR is not required for Tfh-like cells differentiation *in vitro*. (A–C) Naive CD62L^+^CD4^+^ T cells purified from WT and MyoR^−/−^ mice were cultured for 72 h under Th0 and Tfh culture conditions. Expression level of the indicated genes was assessed by quantitative RT-PCR and expressed as relative expression to RPL32 mRNA (A) Aliquots of cells were stimulated for 4 h with PMA and ionomycin and tested for IL-21 production by intracellular staining. Numbers indicate the percentage of IL-21^+^ in each panel (B). Histograms in (C) represent the mean ± SD of IL-21^+^ cells from two independent experiments; (D) In vitro-derived Tfh-like cells from WT and MyoR^−/−^ mice (1,6×10^4^ cells/well) were irradiated and incubated with freshly purified naive B cells from C57BL/6 mice (5×10^5^ cells/well) and anti-CD3 mAbs (5 µg/ml). Culture supernatants of T/B cells were tested on day 7 for IgG1 content by ELISA. Results are expressed as mean of triplicates cultures ± SD and are representative of two independent experiments. Data are representative of at least two independent experiments with similar results. ns, not significant; nd, not detectable

To evaluate whether MyoR is required for the differentiation of Tfh cells, naive CD4^+^ T cells from wild type (WT) and MyoR^−/−^ mice were activated and analyzed as described in the previous section. Upon IL-6-treatment, c-Maf and IL-21 mRNA were similarly induced in Tfh-like cells from both WT and MyoR^−/−^ mice (Figure 3A, middle and right panels). As a control, MyoR mRNA expression was detected in the Tfh culture from WT mice only (Figure 3A, left panel). The secretion of IL-21 by *in vitro* differentiated Tfh-like cells was further investigated by intracellular FACS staining. As shown in Figure 3B, C, and in agreement with the qPCR analysis, the absence of MyoR did not affect the capacity of cells to differentiate into IL-21 producing cells in response to IL-6. Finally, we evaluated the ability of cells lacking expression of MyoR to provide help to wild-type naive B cells *in vitro* when cultured under Tfh-like conditions. The capacity of Tfh cells to promote B cell differentiation into antibody secreting cells was not affected by the absence of MyoR (Figure 3D). Collectively, these data suggest that MyoR is dispensable for the differentiation and function of Tfh-like cells *in vitro*.

### Tfh cells differentiate normally and regulate optimal humoral response in MyoR^−/−^ mice

**Figure 4:**
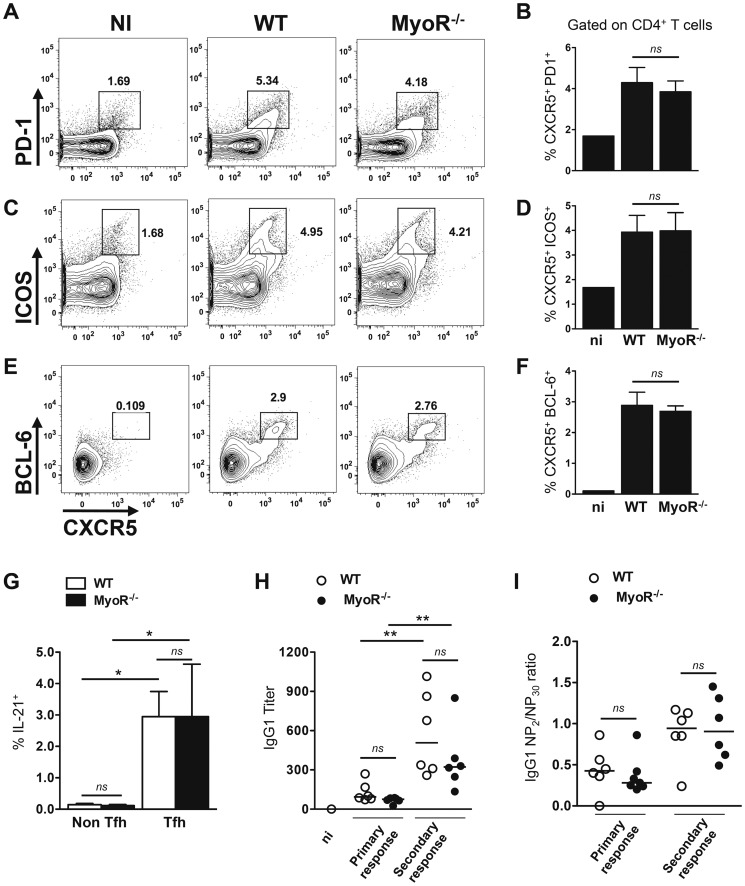
MyoR is dispensable for T-dependent humoral responses. (A–F) WT and MyoR^−/−^ mice were immunized against NP-KLH/Alum (A–D) or KLH/Alum (E, F) and tested for Tfh cell marker expression in the draining lymph nodes. Representative contour plots showed the expression of CXCR5^+^PD-1^+^ (A), CXCR5^+^ICOS^+^ (C) or CXCR5^+^ BCL-6 ^+^ (E) among CD4^+^ T cells. Histograms in (B, D, F) represent the mean ± SD of marker-positive cells from 4 individual mice and are representative of 3 independent experiments. (G) Aliquots of cells were stimulated for 4 h with PMA and ionomycin and tested for IL-21 production by intracellular staining. Histograms represent the mean ± SD of IL-21^+^ cells in the Tfh (CXCR5^+^PD-1^+^) and non-Tfh (CXCR5^−^PD-1^−^) subsets of WT and MyoR^−/−^ mice (4 mice/group). (H) NP-specific serum IgG1 titers of WT (open symbols) and MyoR^−/−^ (closed symbols) mice were determined at different timings after i.p. immunization with NP-KLH/Alum. (I) sera from panel H were tested for NP-affinity. Relative affinities are expressed as ratio of 50% binding on NP_2_-BSA and NP_30_-BSA- coated plates. Each dot represents a mouse. Data are representative of two independent experiments. *: p<0.05; **: p<0.01; ns, not significant; n.i., non immune mouse.

The role of MyoR in the generation of Tfh cells was next tested *in vivo* following immunization of wild type and MyoR^−/−^ mice with KLH/Alum or NP-KLH/Alum in different experimental settings. In the first set of experiments, draining lymph nodes were recovered on day 7 after immunization and analyzed for the induction of Tfh cells according to the expression of CXCR5/PD-1, CXCR5/ICOS and CXCR5/BCL6, as previously reported [38]. Regardless of the staining procedure, we did not find any difference in Tfh cell generation between WT and MyoR^−/−^ mice (Figure 4A–F). Moreover, Tfh cells from both strains of mice secreted equivalent amounts of IL-21, as assessed by intracellular FACS staining (Figure 4G).

In the next set of experiments, control and MyoR^−/−^ mice were inoculated with NP-KLH/Alum and the levels of NP-specific IgG1 antibodies were determined 14 days after primary and secondary immunization. Our data showed that the production of NP-specific IgG1 was not affected by the absence of MyoR either during primary or secondary responses (Figure 4H). Similar results were obtained for NP-specific IgG2a and IgE isotypes (Figure S3).

Affinity maturation is a hallmark of Tfh-dependent antibodies. Control and MyoR-deficient mice were therefore immunized twice with NP-KLH and the relative affinities of the anti-NP IgG1 antibodies were determined by comparing their binding to NP on differentially conjugated BSA carrier proteins, as previously described [39]. The results presented in Figure 4I clearly show that the anti-NP antibodies produced by MyoR^−/−^ mice displayed similar relative affinities to those obtained from control mice, both after primary and secondary immunization, suggesting that MyoR-deficiency did not have significant impact on antibody responses to T-dependent antigens.

## Discussion

Once lymphocyte commitment and/or differentiation are initiated, cells develop mechanisms to reinforce their differentiation program, ultimately ending with epigenetic modifications that lead to the acquisition of a stable and specific phenotype. This is a highly specific and dynamic process accompanied by changes in the expression patterns of thousands of genes at different stages [40], [41]. How specific transcription factors contribute to the functional characteristics of the different T cell types is a topic of great interest in immunology [42], [43].

Recent studies have suggested a role for bHLH proteins in T cell development and function [44]. The bHLH proteins HEB, E12 and E47 are expressed in the thymus, [45]–[48] and mice with a targeted disruption of either the HEB or E2A (coding for both E12 and E47) gene have an early T cell developmental block and ultimately succumb to thymic lymphomas [47], [49]. Moreover, the bHLH protein Dec2 is preferentially expressed in Th2 cells and has been shown to facilitate Th2 differentiation *in vitro* and *in vivo* through the upregulation of interleukin-2 receptor alpha (IL-2Rα) expression [50]. Twist-1 is another bHLH gene induced by STAT4 signaling in Th1 cells that limits the expression of the inflammatory cytokines IFNγ and TNFα [51], [52]. This transcription factor has been also reported to negatively regulate Th17 and Tfh cells differentiation by repressing the expression of IL6-R [53]. bHLH factors play therefore an important role in T cell development, differentiation and lymphomagenesis.

The expression of MyoR was initially suggested to be restricted to the precursor of skeletal muscle lineage [25], but expression of this factor has been documented in a wide variety of tissues. For example, MyoR is highly expressed in adult kidney stem-like side population cells [54] where it plays a functional role in the kidney regeneration process [55]. Our results demonstrated that MyoR mRNA was also expressed in activated T lymphocytes. This is in agreement with QPCR analysis and tissue blot results showing the presence of MyoR transcript in the spleen [55], [56]. We noticed that MyoR was progressively induced during the course of Tfh-like cell differentiation *in vitro*, and reached a peak at the later stage of Tfh differentiation. Comparison with other cell subsets (Th1, Th2, Th17) at a late stage of differentiation revealed a higher expression in Tfh-like cells only. Interestingly, MyoR mRNA was also expressed at higher levels in vaccination-induced Tfh cells *in vivo*, suggesting that MyoR could be a transcription factor associated with this particular Th cell subset.

Despite numerous efforts, we were unable to detect expression of the MyoR protein by *in vitro* generated Th cell populations. However, retroviral-induced MyoR overexpression led to significant accumulation of MyoR protein in activated murine T cells, as monitored by western blot analysis (data not shown), indicating that translation and protein accumulation could occur in this particular cell type. Of note however, virally transduced cells expressed very high levels of MyoR mRNA (over 1000 fold higher relative to IL-6-treated Th cells), suggesting that, in conventional helper T cells subpopulations, the level of MyoR protein expression fall below the detection limit of currently available reagents and techniques.

Given that Tfh-like cells were generated *in vitro* in the presence of IL-6, it is tempting to assume that MyoR could be an IL-6-responsive gene. In agreement with this assumption, MyoR expression was severely impaired in STAT3-deficient Th cells, suggesting that IL-6/STAT3 signaling is required to achieve optimal expression. However, IL-6 alone was not sufficient to promote MyoR mRNA expression in naive Th cells, indicating that other TcR-induced transcription factors might play a role in MyoR transcriptional regulation.

Of note, Th17 cells were induced *in vitro* by a combination of IL-6 and TFGβ. The observation that MyoR mRNA was not upregulated in this Th cell subset suggested that the TGFβ present in the culture environment could prevent MyoR transcription. In agreement with this hypothesis, Li et al recently reported that MyoR was substantially up-regulated in the oviducts of TGFβ receptor-1 (Tgfbr1) conditional KO mice [57]. Collectively, these data suggest that the transcriptional expression of MyoR is a complex process involving both positive and negative regulations.

The expression pattern of MyoR suggested that it might play a role in Tfh cell differentiation and/or function. However, MyoR-deficient CD4 T cells were not impaired in Tfh-like cell differentiation *in vitro* and MyoR^−/−^ mice developed Tfh cells and optimal T-dependent humoral responses *in vivo*. One explanation could be that MyoR function is compensated by other members of the bHLH family in MyoR-KO mice. Consistent with this, it is striking that a complete absence of the major muscles of mastication was observed in double MyoR/capsulin-KO mice, although no head muscle defect was revealed with either single-gene deletion [27]. Thus, given the diversity in the homo-/hetero-dimerizations of the bHLH family members and the functional redundancies among bHLH factors, a full dissection of the MyoR role during Tfh cells differentiation would require the development of genetic models where the effect of the absence of MyoR could be studied in combination with a deficiency in other bHLH family members.

Moreover, although MyoR mRNA expression was clearly associated with Tfh cells responding to alum-precipitated antigens, it is plausible that other types of Tfh responses might be more suited to assess the role of MyoR *in vivo*. For instance, both systemic infections (lymphocytic choriomeningitis virus, LCMV; vesicular stomatitis virus, VSV) and mucosal infections (influenza virus) have been shown to induce a rapid and sustained Tfh cell differentiation that is required to achieve a protective humoral response [58], [59]. Thus, further investigations using virus-infected MyoR-deficient mice might disclose in detail the role of MyoR during Tfh-dependent responses against replicative viruses.

Finally, although the functional significance of MyoR mRNA upregulation during Tfh differentiation remains obscure, expression of this mRNA could be part of the biomarker arsenal defined to identify the Tfh cell subset after *in vitro* or *in vivo* treatments.

## Materials and Methods

### Media and reagents

The medium used throughout this study was RPMI 1640 supplemented with 5% fetal calf serum (FCS), penicillin, streptomycin, glutamine, nonessential amino acids, 1 mM sodium pyruvate and 0.05 mM 2-mercaptoethanol. Anti-CD3 and anti-CD28 mAbs were produced in house.

### Mice and immunization

MyoR-KO mice have been previously described [27]. They were purchased from The Jackson Laboratory (Bar Harbor, ME). Mice were backcrossed nine times on C57BL/6 background in our specific pathogen-free (SPF) facility. C57BL/6 mice were purchased from Harlan Nederland (Horst, The Netherlands).

STAT3^flox/flox^ mice and CD4-CRE mice (both on a C57BL/6 background) were kindly provided by Dr Shizuo Akira (Osaka University, Osaka, Japan) and Dr Geert Van Loo (University of Gent, Gent, Belgium), respectively. STAT3^flox/flox^ mice were mated with CD4-CRE mice to generate T-cell compartment-specific STAT3-deficient mice as described in [60]. All mice were used at 6–12 weeks of age.

Mice were immunized by injecting intraperitoneally 75 µg nitrophenyl-keyhole limpet hemocyanin (NP_25_-KLH, Biosearch Technologies, Novato, CA) with 3 mg of Imject Alum (Thermo Fisher Scientific, Rockford, IL). On day 14, mice received a second injection of NP-KLH in saline. Serum levels of NP-specific antibodies were determined by enzyme-linked immunosorbent assay (ELISA) according to standard procedures using mouse isotype-specific rat monoclonal antibodies (IMEX, Université Catholique de Louvain, Brussels, Belgium). In some experiments mice were immunized with KLH/Alum or NP_25_-KLH/Alum in footpads (10 µg/fp) and draining lymph nodes were analyzed for Tfh cell development on day 7.

### Cell purification

CD62L^hi^CD4^+^ T cells from naive animals were purified by magnetic separation, as previously described [39]. B cells were isolated by negative selection with anti-CD43–coupled magnetic beads (Myltenyi Biotech, Bergisch-Gladbach, Germany). The percentage of purified cell fractions in all experiments ranged between 90% and 98%, as estimated by flow cytometry (not shown).

### Priming of naive T cells

Naive T cells (5×10^5^ cells/well in 24-well plates) were activated for 24–96 hours with plastic-coated anti-CD3 mAb (5 µg/ml) and soluble anti-CD28 mAb (1 µg/ml) and cultured under the following conditions: Th0 (neutral); Th1 (IL-12 [10 ng/mL] and anti–IL-4 mAb [10 µg/mL] eBioscience); Th2 (IL-4 [10 ng/ml] and anti-IFN-γ mAbs [10 µg/ml], eBioscience); Th17 (TGF-β [3 ng/mL] Pre Protech, IL-6 [20 ng/mL], anti-IFN-γ mAbs [10 µg/ml] and anti–IL-4 mAb [10 µg/mL]) and Tfh-like conditions (IL-6 [20 ng/ml], eBioscience).

### B cell help assay

After 48 h of *in vitro* polarization, T cells were recovered, washed and rested 24 h in medium before co-culture for 7 days with purified syngeneic B cells (5×10^5^ cells/well) in the presence of anti-CD3 mAb (500 ng/ml). T cells were irradiated (2000 cGy) before the beginning of the co-culture with B cells to prevent their outgrowth during the 7-day culture, as previously described [39]. The IgG1 antibodies in the supernatants were determined by ELISA.

### Quantitative RT-PCR

Total cellular RNA was extracted from cell lysates by the use of TRIzol reagent, and reverse transcription of mRNA was carried out using Superscript II reverse transcriptase (Invitrogen) according to the manufacturer's instructions. Quantitative PCR was performed using a StepOne Plus system (Applied Biosystems, Foster City, CA) with Maxima SYBR Green/ROX qPCR Master Mix (Thermo Fisher Scientific, Waltham, MA). Quantification (with RPL32 as endogenous housekeeping gene) was done using standard curves.

The primer sequences were: MyoR [5′-CTATGTGCACCCTGTGAACCT-3′ (forward) and 5′-GTTGGCTGCAGAAACGTCTT-3′ (right)]; T-bet [5′-CCTGTTGTGGTCCAA-GTTCA-3′ (forward) and 5′-GAAGGAC-AGGAATGGGAACA-3′(right)]; GATA-3 [5′- CAATGCCTGTGGGCTGTAC-3′ (forward) and 5′-GGGTCTGGATGCCTTCTTTC-3′ (right)]; RORgt [5′-TCTA-CACGGCCCTGGTTCT-3′ (forward) and 5′-ATGTTCCA-CTCTCCTCTTCTCTTG-3′ (right)]; c-Maf [5′AGCAGTTGGTGACCATGTCG-3′ (forward) and 5′-TGGAGATCT-CCTGCTTGAGG-3′(right)]; BCL-6 [5′- GCGAAC-CTTGATCTCCAGTC-3′ (forward) and 5′- CAGGGACCTGTTCACGAGAT-3′(right)]; IL-21 [5′-GCCAGATCGCCTCCT-GATTA-3′ (forward) and 5′-CATGCTCACAGTG-CCCCTTT-3′ (right)].

Levels of mRNA expression were normalized to the Ribosomal Protein L32 (RPL32) mRNA level in each sample.

### Flow cytometry

Specific cell-surface staining was performed using a standard procedure with anti-CD4, anti-PD1, anti-ICOS (eBioscience) and anti-CXCR5 mAbs (BD Pharmingen).

For intracellular cytokine staining, primed cells were restimulated for 4 hours with Phorbol 12-Myristate 13-Acetate (PMA) (50 ng/ml) and ionomycin (250 ng/ml) (Sigma-Aldrich) in the presence of monensin (1/1000) (eBioscience). Cells were fixed and permeabilized with BD Cytofix/Cytoperm kit (BD Pharmingen) and stained in a two-step procedure using recombinant mouse IL-21R subunit - human Fc chimera (R&D Systems, Minneapolis, MN) followed by phycoerythrin (PE)-conjugated anti-human IgG (Jackson ImmunoResearch, West Baltimore Pike, PA).

Intracellular Bcl-6 staining was performed with a PE-conjugated monoclonal antibody to Bcl-6 (clone mGlI191E, eBioscience) and according to the FoxP3 staining set protocol (eBioscience).

Cells were analyzed by flow cytometry with a FACS Canto II (BD Biosciences) and analyzed using FlowJo Software.

### Statistical analysis

All statistical tests were performed using Prism5, and differences between groups were analyzed with the Mann-Whitney test for two-tailed data. A p-value of less than 0.05 was considered as significant.

### Ethics information

The experiments were carried out in strict accordance with the relevant laws of the country and with institutional guidelines. We received specific approval for this study from the Université Libre de Bruxelles Institutional Animal Care and Use Committee (protocol number CEBEA-5)

## Supporting Information

Figure S1 **Endogenous MyoR protein is not detectable is Tfh-like cells.** (A) Naive CD62L^+^CD4^+^ T cells from WT mice were stimulated as described in Fig.1 After 96 h of culture, total lysates were harvested and immunoblot was performed for MyoR (Sc-Cruz-9556); beta-actin (A2066, Sigma) was used as loading control. 293T cells transfected with a plasmid coding for MyoR was used as positive control. Data are representative of three independent experiments. (B) The cells used in (A) were examined for the expression of MyoR by RT-PCR. Histograms represent mean ± SD of duplicates.
(TIF)

Figure S2 **IL-21 induces MyoR mRNA** ***in vitro*** **.** Naive CD62L^+^CD4^+^ T cells purified from WT mice were stimulated for 48 h and 72 h with plastic-coated anti-CD3 and anti-CD28 mAbs under neutral conditions (medium), in the presence of IL-6 or IL-21. Expression level of MyoR was assessed by quantitative RT-PCR and expressed as relative expression to RPL32 mRNA. Histograms represent the mean ± SD of duplicates.
(TIF)

Figure S3 **MyoR is dispensable for IgG2a and IgE antibody response.** WT and MyoR^−/−^ mice were tested for NP-specific IgG2a (A) and IgE (B) upon secondary immunization (day 14) with NP-KLH/Alum. Each dot represents a mouse. ns, not significant.
(TIF)

Table S1 **Microarray analysis of gene differentially expressed among in vitro-derived Th0 and Tfh-like cells.** Results are expressed as log2 transformed ratio of spot intensity between Th0 and Tfh-like cells for each gene. MyoR is indicated in blue.
(XLSX)
