# Correction: Myor/ABF-1 Mrna Expression Marks Follicular Helper T Cells but Is Dispensable for Tfh Cell Differentiation and Function *In Vivo*

**DOI:** 10.1371/annotation/b0c3d5f5-5778-48af-85ed-abf1b9113a48

**Published:** 2014-01-13

**Authors:** Delphine Debuisson, Nathalie Mari, Sébastien Denanglaire, Oberdan Leo, Fabienne Andris

The term "mRNA" is incorrectly capitalized in the article title. The correct title is: 

"Myor/ABF-1 mRNA Expression Marks Follicular Helper T Cells but Is Dispensable for Tfh Cell Differentiation and Function *In Vivo*."

The correct Citation is: Debuisson D, Mari N, Denanglaire S, Leo O, Andris F (2013) Myor/ABF-1 mRNA Expression Marks Follicular Helper T Cells but Is Dispensable for Tfh Cell Differentiation and Function *In Vivo*. PLoS ONE 8(12): e84415. doi:10.1371/journal.pone.0084415.

