# Supplementary figures and images for: Myor/ABF-1 Mrna Expression Marks Follicular Helper T Cells but Is Dispensable for Tfh Cell Differentiation and Function In Vivo
Source: PLoS One. 2013 Dec 26;8(12):e84415. doi: 10.1371/journal.pone.0084415 (PMC3873420; doi:10.1371/journal.pone.0084415)

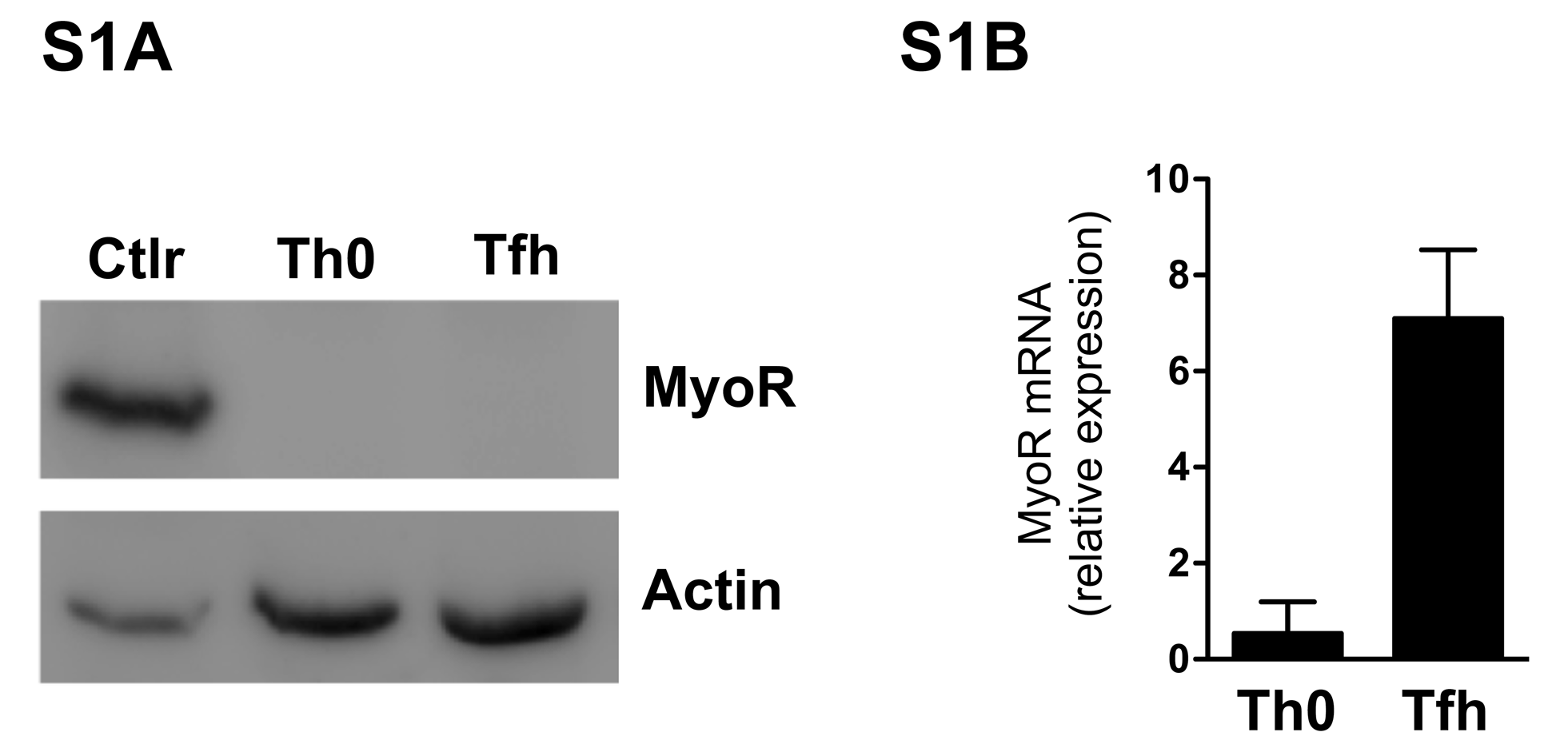

Supplement: Figure S1 — Endogenous MyoR protein is not detectable is Tfh-like cells. (A) Naive CD62L+CD4+ T cells from WT mice were stimulated as described in Fig.1 After 96 h of culture, total lysates were harvested and immunoblot was performed for MyoR (Sc-Cruz-9556); beta-actin (A2066, Sigma) was used as loading control. 293T cells transfected with a plasmid coding for MyoR was used as positive control. Data are representative of three independent experiments. (B) The cells used in (A) were examined for the expression of MyoR by RT-PCR. Histograms represent mean ± SD of duplicates. (TIF) [file pone.0084415.s001.tif]

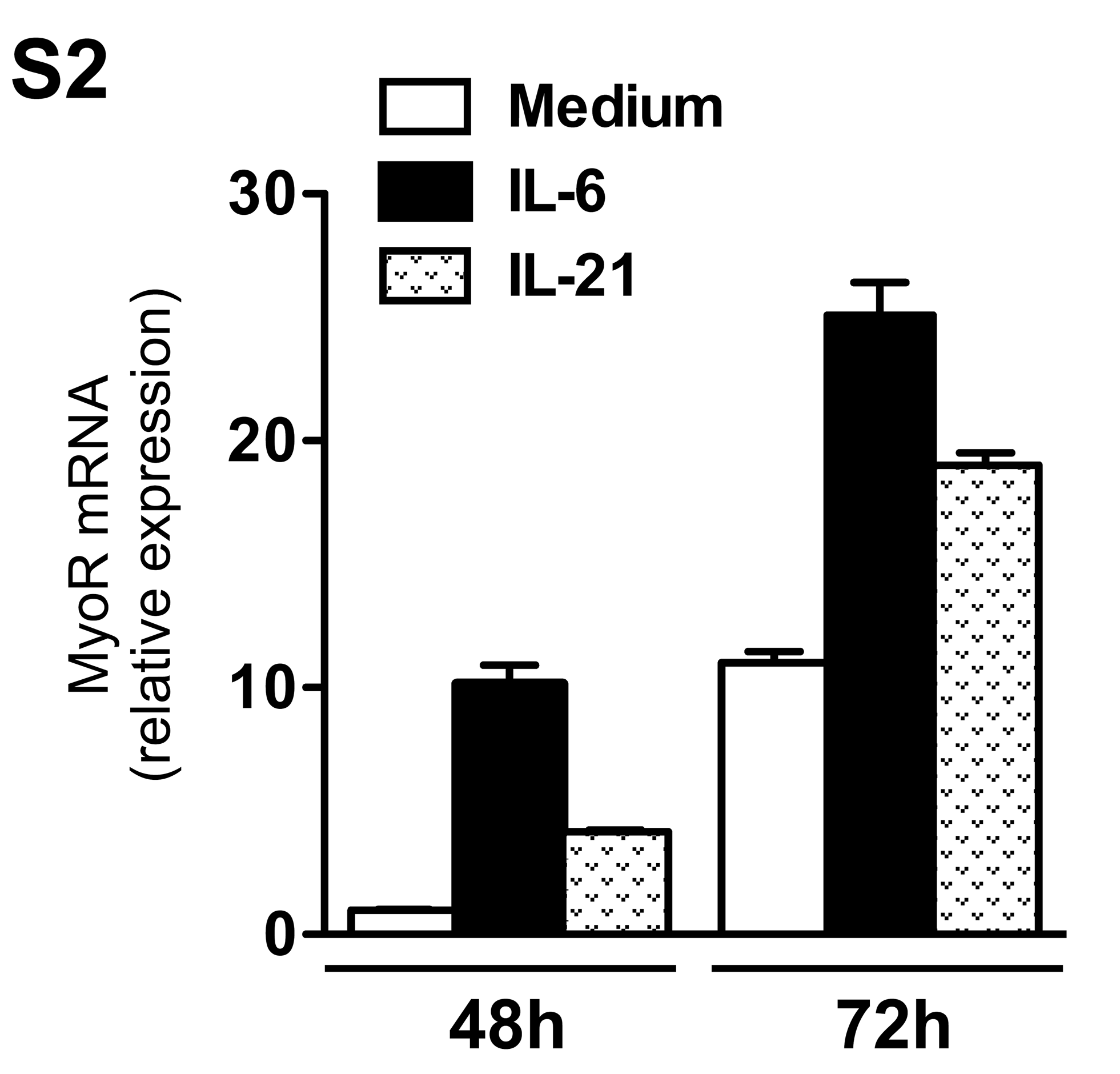

Supplement: Figure S2 — IL-21 induces MyoR mRNA in vitro . Naive CD62L+CD4+ T cells purified from WT mice were stimulated for 48 h and 72 h with plastic-coated anti-CD3 and anti-CD28 mAbs under neutral conditions (medium), in the presence of IL-6 or IL-21. Expression level of MyoR was assessed by quantitative RT-PCR and expressed as relative expression to RPL32 mRNA. Histograms represent the mean ± SD of duplicates. (TIF) [file pone.0084415.s002.tif]

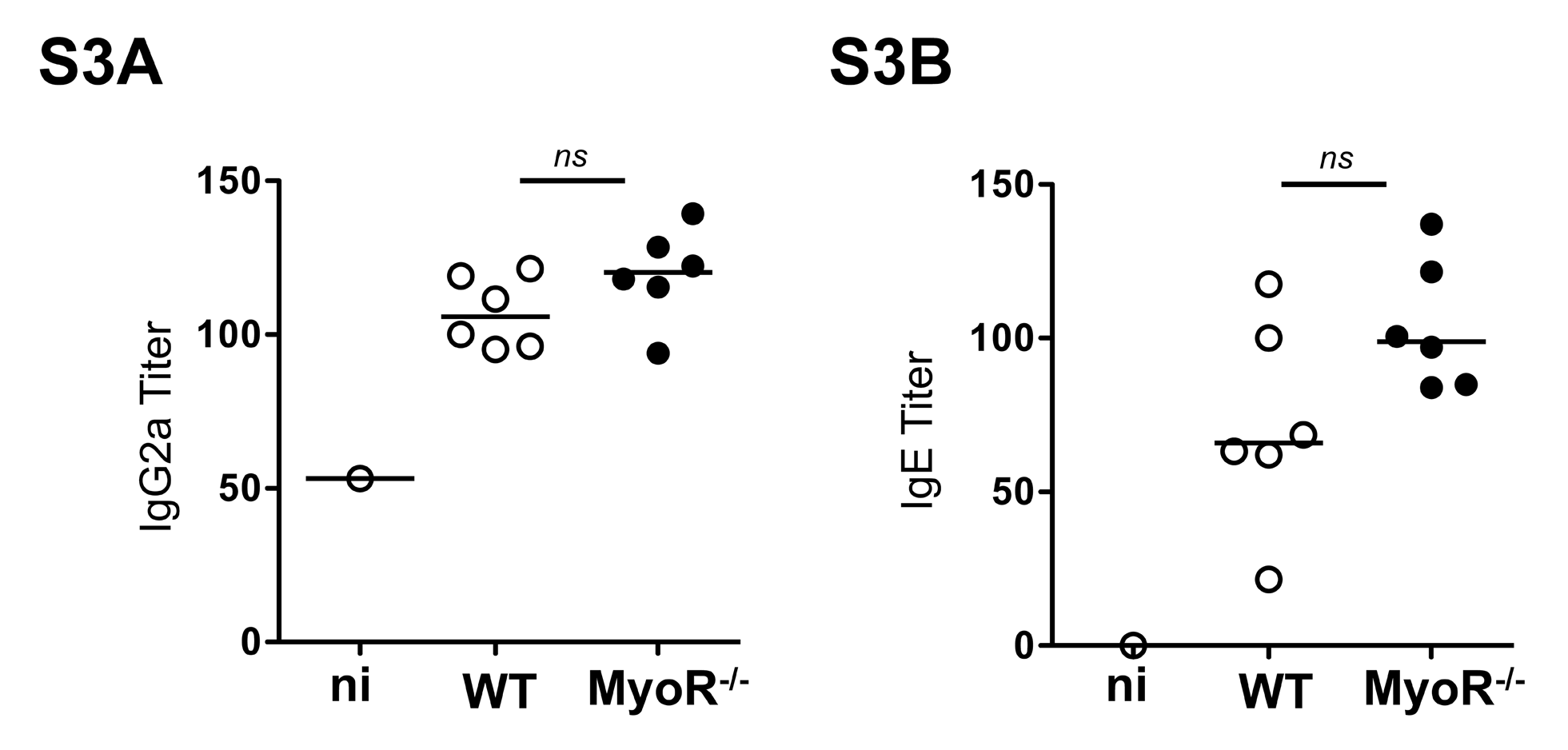

Supplement: Figure S3 — MyoR is dispensable for IgG2a and IgE antibody response. WT and MyoR−/− mice were tested for NP-specific IgG2a (A) and IgE (B) upon secondary immunization (day 14) with NP-KLH/Alum. Each dot represents a mouse. ns, not significant. (TIF) [file pone.0084415.s003.tif]
